# Major Cognitive Changes and Micrographia following Globus Pallidus Infarct

**DOI:** 10.1155/2014/252486

**Published:** 2014-11-12

**Authors:** Sarah Nelson, Hassanain Toma, Haley LaMonica, Tinatin Chabrashvili

**Affiliations:** ^1^Massachusetts General Hospital, 55 Fruit Street, Boston, MA 02114, USA; ^2^Royal Columbian Hospital, 330 E Columbia Street, New Westminster, BC, Canada V3L 3W7; ^3^Tufts Medical Center, 800 Washington Street, Boston, MA 02111, USA

## Abstract

*Importance*. Globus pallidus (GP) lesions are well known to cause motor deficits but are less commonly—and perhaps not conclusively—associated with cognitive problems. *Observations*. We present a 45-year-old male with no significant neurological or psychological problems who after suffering a GP infarct was subsequently found to have substantial cognitive problems and micrographia. Formal neuropsychological testing was not possible due to lack of patient follow-up. *Conclusions and Relevance*. Despite the conflicting literature on the association of GP lesions and cognitive deficits, our patient demonstrated significant neuropsychological changes following his stroke. In addition, evidence of micrographia likely adds to the literature on the localization of this finding. Our case thus suggests that neuropsychological testing may be beneficial after GP strokes.

## 1. Case Report

**Figure 1:**
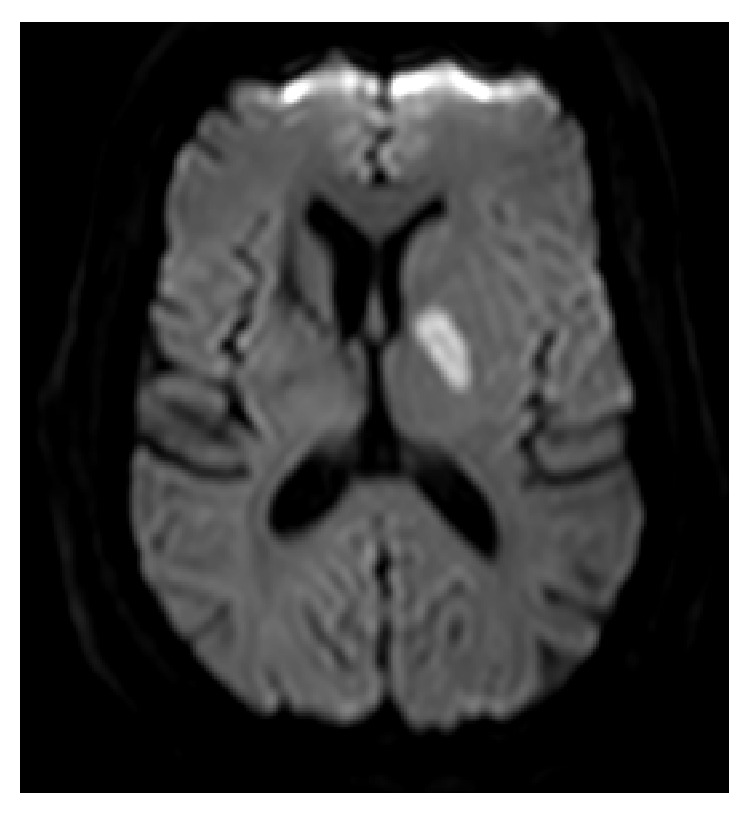
Axial MRI of the brain. Diffusion weighted imaging sequence showing acute left globus pallidus infarct.

A 45-year-old right-handed male with a history of hypertension, diabetes mellitus, and depression—but no other known cognitive issues—presented in November 2013 with altered mental status. He had been found stuck in a basement alcove for an unknown period of time prior to being brought to the emergency room by emergency medical services, and the patient was unable to provide an accurate timeline of events leading up to his presentation. On neurologic examination in the emergency room, he was slow to respond to questions and did not know the correct date. Right-sided weakness but no Parkinsonian features or obvious changes in tone were noted. Magnetic resonance imaging (MRI) of the brain demonstrated a left acute to subacute GP infarct and an old right GP lacunar infarct (Figure 1). Magnetic resonance angiogram of the head and neck was normal. Basic laboratories on admission were generally unremarkable except for glucose 438 mg/dL, creatinine 1.45 mg/dL, and a white blood cell count of 13 K/uL; all of these values normalized during his hospital stay. A serum toxicology screen on admission was unremarkable. Low-density lipoprotein was 160 mg/dL and hemoglobin A1c was 12.0%. Transthoracic echocardiogram showed a normal ejection fraction and no interatrial shunt. Telemetry showed no signs of arrhythmia.

The patient was started on aspirin and atorvastatin as well as medications to control his blood pressure and diabetes. On discharge two days later, he exhibited mild right-sided weakness and slight problems in orientation; he knew that he was in the hospital but did not know its name and could not state the exact date in November 2013.

However, five days later the patient again presented with altered mental status. He was oriented to self only, produced only one-word answers with considerable prompting, and appeared abulic. Overall, his family felt he was a completely different person than prior to his stroke. Repeat MRI of the brain showed interval evolution of the left BG infarct but no other new findings. No other causes for his exam findings (including seizure, infection, or toxic-metabolic etiologies) were discovered. He was discharged in early December 2013 on similar medications as on his prior admission.

**Figure 2:**
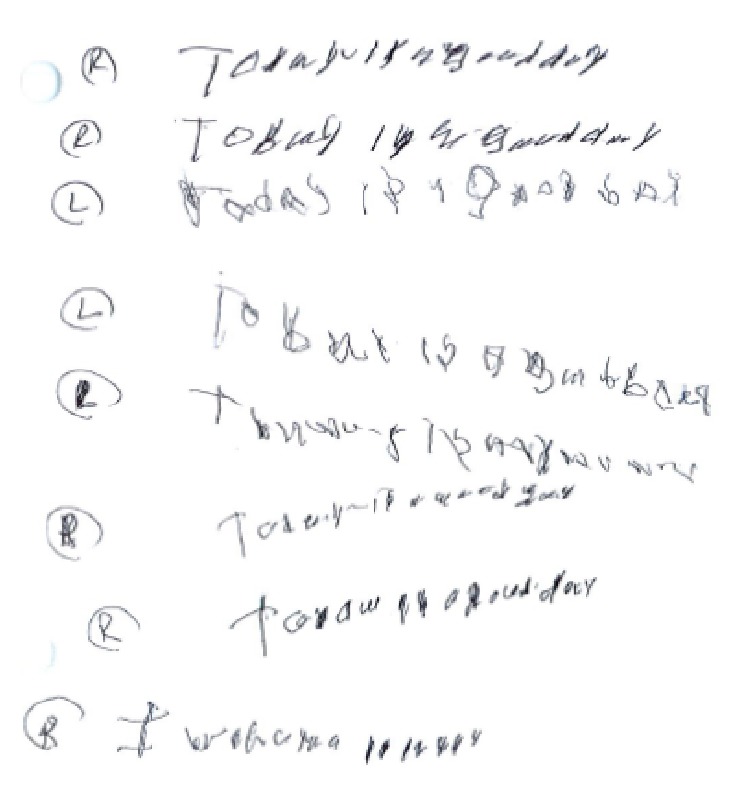
Handwriting samples. Micrographia was primarily demonstrated in the patient's right hand (designated “R”) as compared to his left hand (designated “L”).

The patient did not follow up for formal neuropsychological tests, but he had undergone bedside testing during the latter half of his second hospital stay. Deficits in the Montreal Cognitive Assessment were present in all domains but were most prominent in short-term and working memory, visuospatial ability, executive function, attention, and concentration (Table 1). He perseverated in the alternate triangles and squares sequencing task. Expressive aphasia was evident while describing the Boston Diagnostic Aphasia Examination's Cookie Theft picture. Micrographia, primarily with his right hand, was also present (Figure 2).

In a follow-up visit with his primary care physician 3 days after discharge from the second hospital admission, he was noted to be oriented to person and date, but not to location. The patient displayed language and comprehension deficits: he was “unable to follow commands to fully assess strength and rarely speaks in full sentences but can answer simple sentences with great effort.” However, more rigorous testing of other aspects of his cognition was not performed.

## 2. Discussion

GP lesions are typically associated with motor deficits, including Parkinsonism, tremor, and dystonia [1, 2]. However, somewhat controversially and undoubtedly less commonly, these lesions may also be associated with cognitive disorders and micrographia.

Many of the studies evaluating cognitive problems following GP lesions derive from the Parkinson's disease (PD) literature. Following pallidotomy or other surgical lesioning of the GP in PD, various cognitive changes occurred in two studies including abulia, mental automatism, depression, and deficits in attention, naming, encoding, retrieval, and motor learning and speed [3, 4]. In one study, globus pallidus interna (GPi) lesion location along anteromedial to posterolateral axis was associated with both cognitive and motor postsurgical outcome [5]. In a review, 5 of 16 studies on GPi-deep brain stimulation (DBS) reported cognitive changes such as mania, hypomania, suicide, and improved depression. Eleven out of 25 studies regarding pallidotomy described behavior problems including depression, transient confusion, hallucinations, psychotic depression, and paranoia [6].

Specific to the stroke literature, cognitive effects after GP infarction include executive function problems, verbal memory problems, abulia, loss of emotional expression, reduction of spontaneous thought content, disinhibition, and obsessive-compulsive disorders [8, 9]. Small subcortical lesions involving the thalamus, caudate, and GP appear to disrupt cortico-subcortical circuits, resulting in cognitive dysfunction [10]. In yet other literature on GP lesions due to other reasons, worsening general cognition, immediate recall problems, and improvement in executive function were demonstrated following GPi lesions [2, 11, 12].

Conversely, other literature has shown no association between the GP and cognition. For example, several studies showed no significant cognitive changes after pallidal DBS in PD patients [13–16]. Similarly, there were generally no neuropsychological changes after pallidotomy in many studies [17–21]. A meta-analysis of 240 patients with caudate, putamen, and GP lesions due to various causes revealed that motor deficits were frequently associated with lentiform nucleus lesions. Few behavioral abnormalities were associated with GP lesions and only with bilateral lesions [1].

Furthermore, our patient displayed evidence of micrographia. Small handwriting is a signature feature of Parkinson's disease, which is caused by loss of dopaminergic neurons in the substantia nigra pars compacta. As shown in the small body of literature on micrographia associated with stroke, localizations of this symptom following stroke have included frontal lesions (e.g., anterior cerebral artery infarctions), left thalamomesencephalic region, and BG [22–28]. One study demonstrated that micrographia could in fact indicate the presence of a stroke [27]. Another article's authors emphasized that, up until their article, the left BG-thalamic region was involved in all reported cases of micrographia after subcortical infarction [28]. Most relevant to our patient, four cases of micrographia were due to strokes in the lenticular nucleus [29]. In addition, other GP lesions have been found to cause micrographia, including GPi-DBS, a finding that was thought to be a manifestation of hypokinesia due to disturbance in pallido-thalamo-cortical outflow pathways [30].

A couple of weaknesses of our study should be addressed. Although the left BG infarct was associated with significant cognitive impairment and micrographia in our patient, we cannot exclude the possibility that lesions in both basal ganglia were required to generate these findings since our patient also had a chronic right BG infarct. Another weakness is that it is possible that, due to the resolution of our patient's MRI, involvement of the anterior portion of the thalamus may not have been definitively excluded. As noted in the literature, various and significant cognitive changes including apathy, word-finding difficulties, perseveration, superposition of unrelated information, and memory problems can also occur after thalamic strokes [31–33].

The literature is conflicting on whether cognitive effects are associated with GP lesions. However, after suffering a GP infarct, our patient demonstrated major cognitive deficits, particularly in the domains of short-term and working memory, visuospatial ability, executive function, attention, and concentration. Our patient also displayed evidence of micrographia, though we were unable to further evaluate this finding because the patient was lost to follow up. BG region strokes appear to be associated with micrographia, albeit infrequently. Our case emphasizes that GP lesions can cause significant cognitive sequelae and likely adds to our understanding on the localizations of micrographia. Neuropsychological testing should be considered in patients suffering from GP strokes.

## Figures and Tables

**Table 1:** Montreal Cognitive Assessment cognitive domains (derived from [7]).

Cognitive domain	Patient's score	Normal score
Short-term memory	0	5
Visuospatial ability	1	4
Executive function^*^	0	4
Attention/concentration/working memory	2	6
Language^*^	4	6
Orientation	5	6
Total	**12**	**31**

^*^Because the phonemic fluency task (1 point) tests both executive function and language, the total number of possible points in this table is 31.
